# Characterizing changes in abdominal aortic aneurysms using principal wall strain ultrasound elastography

**DOI:** 10.3389/fcvm.2025.1613881

**Published:** 2025-09-15

**Authors:** Baqir J. Kedwai, Zachary R. Zottola, Daniel J. Lehane, Joshua T. Geiger, Micheal C. Stoner, Michael S. Richards, Doran S. Mix

**Affiliations:** ^1^ Division of Vascular Surgery, University of Rochester Medical Center, Rochester, NY, United States; ^2^ Department of Biomedical Engineering, Rochester Institute of Technology, Rochester, NY, United States

**Keywords:** aorta, ultrasound, AAA, vascular surgery, elastography

## Abstract

**Introduction:**

Aortic principal wall strain is a biomechanical parameter correlated with aneurysm growth rate that affects abdominal aortic aneurysm (AAA) stability. Characterize changes in pressure-normalized maximum mean aortic principal wall strain $\left(\bar{\varepsilon_{\rho +}}/\mathrm{PP}\right)$ using ultrasound elastography (USE).

**Methods:**

Axial ultrasound images of patient AAAs were collected at two consecutive clinic visits. The $\bar{\varepsilon_{\rho +}}/\mathrm{PP}$ for each image was calculated using a novel finite element mesh technique. The cohort was separated by index $\bar{\varepsilon_{\rho +}}/\mathrm{PP}$ terciles, and the rate of strain change, growth, intervention, and rupture were compared.

**Results:**

31 patients with a median age of 72.0 [65.0, 77.5] at index visits were included, with follow-up imaging taken at an average interval of 6.2 [6.0, 8.3] months. For the whole cohort, maximum $\bar{\varepsilon_{\rho +}}/\mathrm{PP}$ decreased from 2.1 [1.1, 2.7] %/mmHg to 1.9 [1.3, 2.6] %/mmHg (*p* = 0.08), and maximum AAA diameter increased from a median of 4.3 [4.0, 4.7] cm to 4.4 [4.1, 4.9] cm (*p* = 0.04). The “high-strain” tercile was associated with a strain reduction of −1.3 [−2.5, −1.1] %/mmHg between index and follow-up imaging, as compared to the “low-strain” (−0.1 [−0.6, 0.5] %/mmHg, *p* < 0.01) and “intermediate-strain” (−0.4 [−0.5, −0.3] %/mmHg, *p* = 0.04) terciles. There was no difference in the rate of AAA growth, intervention, or rupture between terciles.

**Discussion:**

The present findings indicate that $\bar{\varepsilon_{\rho +}}/\mathrm{PP}$ at baseline predicts the degree and direction of $\bar{\varepsilon_{\rho +}}/\mathrm{PP}$ change in AAAs over time. These findings offer insight into the natural history of AAA tissue mechanics and demonstrate the potential for a novel ultrasound technique to quantify biomechanical changes in the aortic wall. These findings may aid in the development of patient-specific risk stratification tools informed by biomechanical data in addition to conventional size-based criteria.

## Introduction

Over one million adults in the United States are estimated to have an abdominal aortic aneurysm (AAA) (1). Clinically defined as a regional dilation of the abdominal aorta greater than 50% or a maximum aortic diameter ≥3 cm, this disease process is largely asymptomatic while the aneurysm grows (1–3). AAAs have a significant risk of rupture, associated with an 80% mortality rate (4). This fatal presentation is the leading cause of mortality in the U.S., with 4,500 deaths per year (2).

This disease process is typically silent until catastrophic rupture. Therefore, appropriate management for patients with an AAA depends on timely diagnosis and serial monitoring (4–8). Ultrasound imaging has emerged as a convenient and cost-effective diagnostic tool for AAAs and is validated as an accurate and reliable method for screening (1, 9–11). There is extensive literature supporting the utility of AAA diameter and AAA growth rate as corollaries for rupture risk (12–15). As such, ultrasound-based morphometric analyses currently guide clinical decision-making. The Society for Vascular Surgery (SVS) recommends surgical intervention for women with AAA diameter ≥5.0 cm and men ≥5.5 cm, interval growth ≥0.5 cm in 6 months, or growth ≥1 cm in 1 year (1).

These screening thresholds are effective, but a sizable burden of ruptured AAAs on the U.S. healthcare system remains. Many patients experience rupture below the established size and growth thresholds, while others remain asymptomatic far beyond. It is estimated that 43% of fatal ruptures between 1999 and 2016 did not meet screening criteria for intervention (16).

The study of aortic biomechanics seeks to understand the tissue properties and hemodynamic conditions contributing to AAA degeneration, tissue failure, and rupture. Modern approaches to studying aortic tissue mechanics employ advanced computational techniques and non-invasive *in vivo* imaging technology to accurately incorporate complex geometrical and heterogeneous tissue data into their assessments (17–20). These studies have shown that high wall stress due to pathologic changes in strain and elastic modulus (wall stiffness) predict aneurysm rupture based on the mathematical relationship *stress = elastic modulus × strain* (17–23).

Niestrawska et al. combined mechanical testing with histologic and structural data to develop a three-stage model for the histopathologic progression of AAAs (24). In this model, there is (1) a loss of stiffness and dilation of the aortic wall due to degradation of the extracellular matrix (ECM) and elastic lamina; (2) an increase in aortic wall compliance in the setting of inflammatory cell infiltrate; (3) gradual stiffening of the aneurysm as inflammatory collagen deposition forms a thick “neo-adventitia”. This model categorizes stage 3 aneurysms into one of two phenotypes. The first is the “stable aneurysm” phenotype, in which a thick, protective collagen neo-adventitia forms with minimal inflammation and adipocyte infiltration. The second is the “vulnerable aneurysm” phenotype, which demonstrates persistent inflammatory cell and adipocyte infiltration in the wall.

Mix et al. have developed an ultrasound elastography (USE) technique that utilizes a novel non-rigid image-based registration algorithm to evaluate axial and circumferential strain data from B-mode ultrasound images, then used to calculate patient-specific values of maximum mean principal wall strain $\left(\bar{\varepsilon_{\rho +}}\right)$ normalized to patient pulse pressure (PP), a unit denoted as $\bar{\varepsilon_{\rho +}}/\mathrm{PP}$ (25–27). Zottola et al. applied this novel USE technique to measure the aortic wall $\bar{\varepsilon_{\rho +}}/\mathrm{PP}$ of 113 patients with AAAs. They found that patients with AAAs demonstrating “intermediate” $\bar{\varepsilon_{\rho +}}/\mathrm{PP}$ values between 0.0251 and 0.038%/mmHg were associated with increased AAA growth rate (27). Zottola et al. hypothesized that “intermediate strain” AAAs in their study comprised the vulnerable phenotype of aneurysms, experiencing a rapid growth rate due to continued inflammation and increasing wall compliance rather than forming a stiff neo-adventitia (24–27). Their findings highlight the need to understand the natural history of AAA tissue mechanics as a prerequisite to developing biomechanical parameters that can effectively risk-stratify patients.

Research on the dynamic changes in aortic wall biomechanics over time is limited. Derwich et al. imaged patients over an average 24.5-month follow-up period with a 3D speckle tracking technique to measure changes in mean circumferential aortic strain (MCS) (28). They found that MCS increased independently of AAA diameter over time, though there was no significant change in peak circumferential strain. A subgroup analysis revealed two cohorts of aneurysms: those that experienced an increase in MCS and a decrease in spatial heterogeneity and those with no change in MCS and increasing spatial heterogeneity. The authors hypothesized that these subgroups were consistent with the “stable” and vulnerable phenotypes that Niestrawska et al. had described (28).

The natural history of AAA wall biomechanics is poorly understood, and given the limitations of current AAA screening tools, there is a critical need to explore biomechanical markers that reflect aneurysm progression. The present study aimed to characterize changes in $\bar{\varepsilon_{\rho +}}/\mathrm{PP}$ over time using the USE technique described by Mix et al. and Zottola et al. (25–27). The hypothesis was that the changes in $\bar{\varepsilon_{\rho +}}/\mathrm{PP}$ would differ between AAA strain terciles, with aneurysms experiencing an increase or decrease in $\bar{\varepsilon_{\rho +}}/\mathrm{PP}$ based on their progression along the proposed natural history of histopathologic aneurysmal degeneration. The findings of this study may inform the development of biomechanical parameters to predict AAA rupture risk accurately.

## Materials and methods

**Figure 1:**
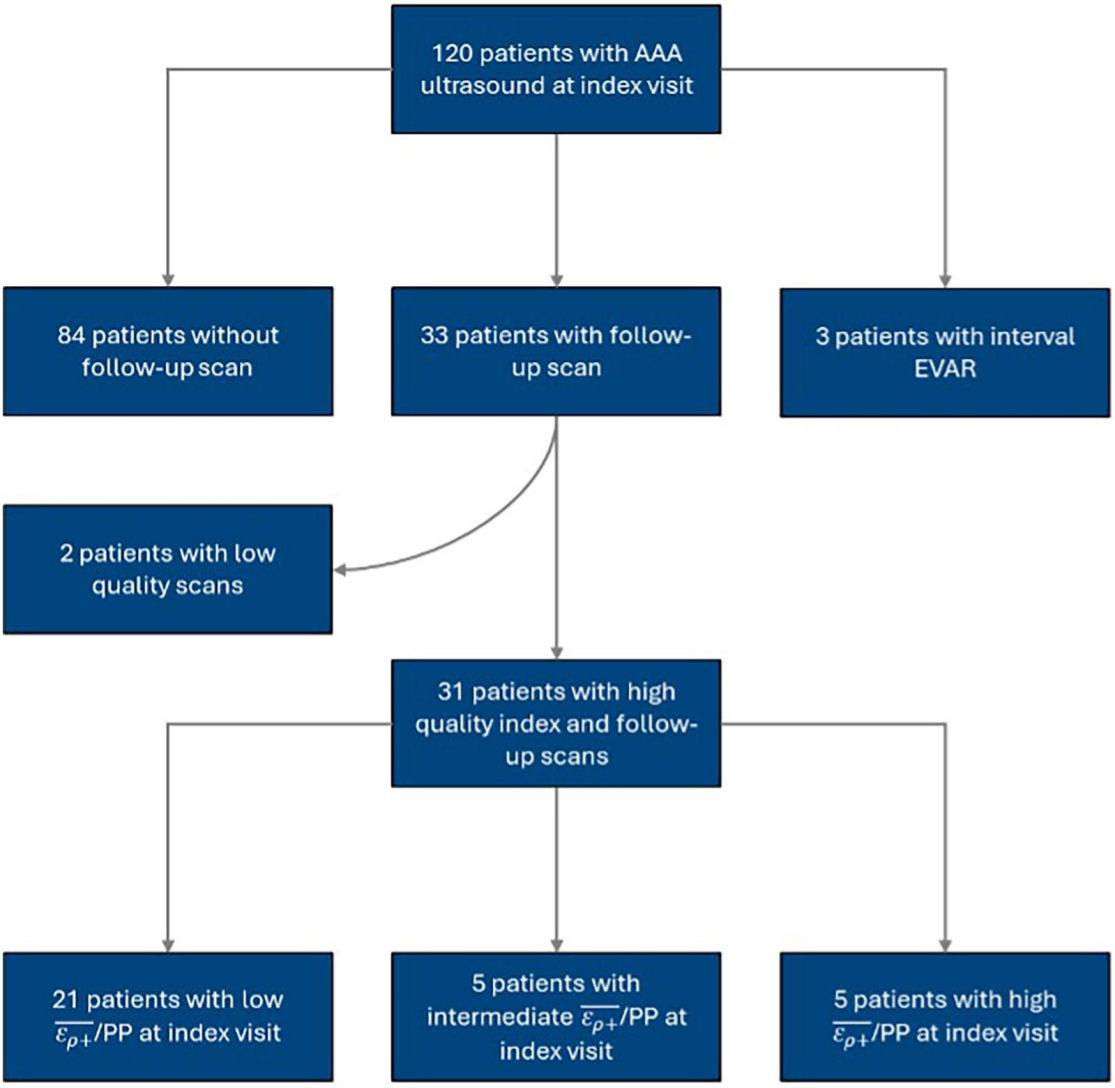
Flow diagram depicting the process for cohort selection.

The present study is a retrospective cohort study of prospectively collected US data of patients with AAAs at the University of Rochester Medical Center (URMC) between 2015 and 2016. Patients were recruited for their baseline or “index” US scan if they were older than 18 with known, unrepaired AAAs. The exclusion criteria consisted of patients without follow-up imaging and patients who underwent surgical repair before follow-up. Index and follow-up US scans were visually inspected and screened for image quality. The cohort was divided into terciles based on the $\bar{\varepsilon_{\rho +}}/\mathrm{PP}$ measured at the index visit, using cutoff values of 0.0251% and 0.038%/mmHg that Zottola et al. identified in their study of the same patient cohort (27). The cohort selection process is illustrated in Figure 1. Additional clinical and imaging data was collected from patients' electronic records. The URMC Research Subjects Review Board reviewed and approved the study and informed consent process.

The authors have previously developed and reported on a novel USE imaging technique that derives strain data by estimating aortic wall deformation over one cardiac cycle using a non-rigid image-based registration algorithm (25–27). In short, this technique uses single-focus, RF ultrasound images to measure and accumulate 2D displacement fields of the aorta in a cross-section, localized at the maximum diameter of the aneurysm. A non-rigid registration technique is used to maintain measurement accuracy over the cardiac cycle. The total accumulated principal strain is calculated from the displacement measurements corresponding to the frames of minimum diastolic to maximum systolic pressure. These principal strain values are then normalized by an independently measured pulse pressure, acquired using a brachial pressure cuff, to obtain our quantitative metric of vessel stiffness.

For the present work, USE imaging was conducted at the vascular surgery outpatient clinic using the Ultrasonix Sonix-Tablet (B.K. Medical, Burlington, MA) or Ultrasonix Sonix-Touch US systems and an Ultrasonix C7-3/50 convex transducer. Axial B-mode images of the aneurysm were taken at the point of maximal diameter. All ultrasound images were captured at a frequency of 5 MHz. Sector and depth settings were adjusted to achieve a recorded frame rate ≥50 frames per second. Image gain was adjusted per user judgment. Patients maintained a 10-second breath-hold during image collection to reduce motion artifacts. The scans were stored as radiofrequency (R.F.) data. Manual blood pressure measurements collected during the clinic encounter were recorded to calculate the patient's pulse pressure at the time of the scan. The authors’ MATLAB algorithm processed R.F. data for each image (2019b, Natick, Massachusetts, MathWorks Inc., RRID: SCR_001622) to calculate $\bar{\varepsilon_{\rho +}}$. This USE algorithm has been validated and described in detail by Mix et al. in previous studies (25, 26).

**Figure 2:**
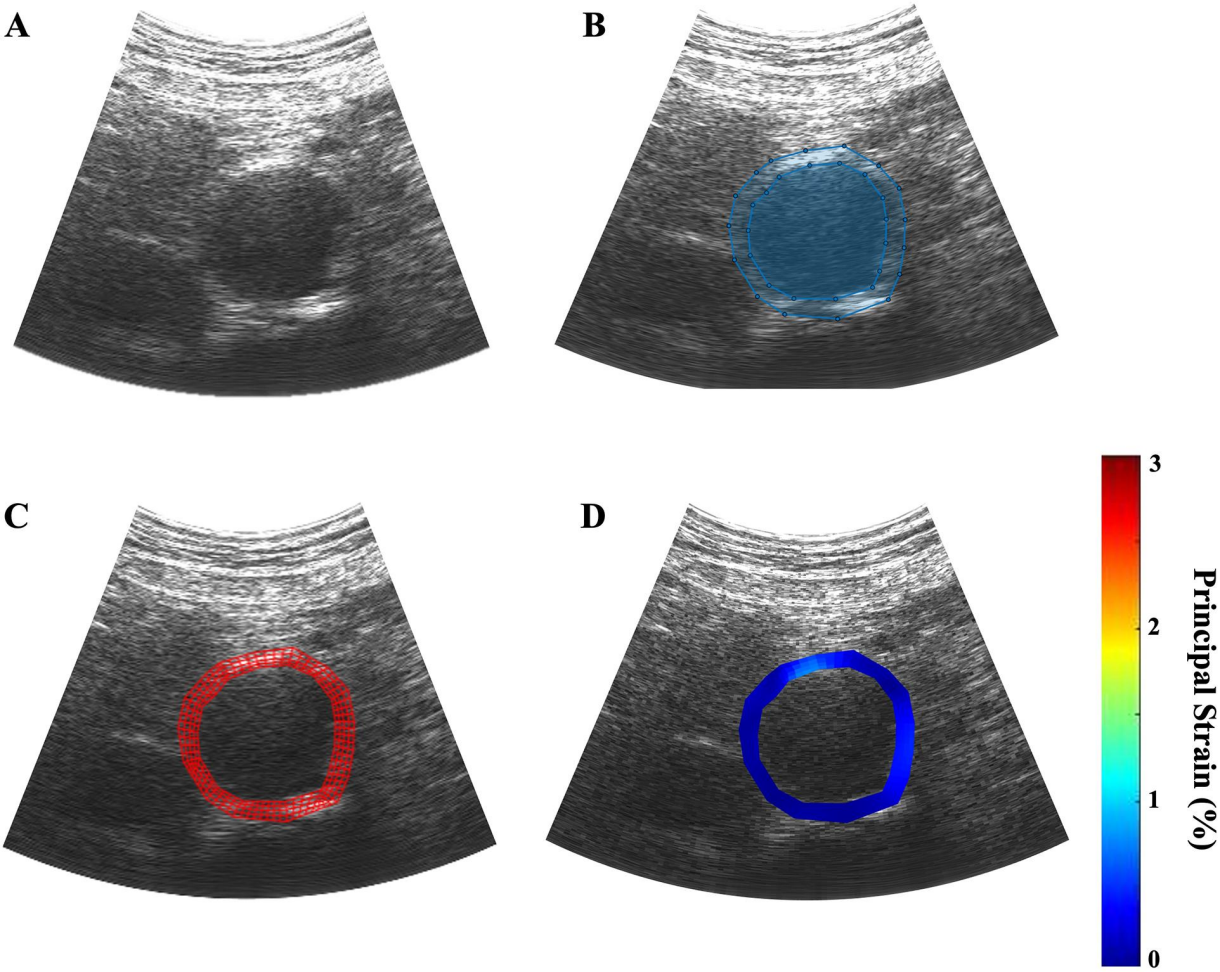
Example of the ultrasound elastography algorithm applied to a patient scan, dimensions: 1,920 × 963 pixels. **(A)** a B-mode ultrasound cine loop of a patient's AAA is captured over one cardiac cycle; **(B)** the inner and outer walls of the aortic aneurysm are manually identified; **(C)** four-node finite element discretization is applied over the user-defined region of interest; **(D)** parametric image is generated to visually depict the regions of variable strain over on cardiac cycle.

A trained reviewer visually analyzed all RF files as B-mode cine loops and identified frame ranges spanning exactly one cardiac cycle from end-diastole to end-diastole. The first frame was then manually segmented by the reviewer to define a polygonal region of interest (ROI) that included the inner and outer aorta and delineated the boundaries between aortic tissue from the lumen and the external environment (Figure 2A). A four-node, quadrilateral finite element discretization was applied and placed over the segmented ROI (Figure 2B). A non-rigid image registration-based displacement estimation algorithm was used to track the frame-to-frame displacement of each element over the cardiac cycle (26).

The mean average displacement of each element was used to calculate the mean average principal wall strain in each frame. The strain measurements of each frame in the cardiac cycle were graphed to visualize and identify the point of $\bar{\varepsilon_{\rho +}}$ in the selected cardiac cycle (Figure 2C). Parametric imaging was applied to the B-mode images to visualize the dynamic changes in strain during the cycle (Figure 2D). The size of the AAA was determined by measuring from outer wall to outer wall at the aneurysm's maximum diameter in the axial plane at the end of the diastole.

The primary outcome was net change in $\bar{\varepsilon_{\rho +}}/\mathrm{PP}$, rate of $\bar{\varepsilon_{\rho +}}/\mathrm{PP}$ change (%/mmHg/year), and growth rate (cm/year) between index and follow-up visits by strain tercile. Secondary outcomes included growth rate and rate of rupture or intervention within 1 and 5 years of index scan. Differences in patient characteristics between the index and follow-up scans were assessed using Fisher's exact tests and Mann–Whitney *U* tests as appropriate. Spearman's rank correlations were used to test for linear associations between the rate of strain change with index strain, index AAA diameter, and AAA growth rate.

The cohort was divided into terciles based on the $\bar{\varepsilon_{\rho +}}/\mathrm{PP}$ values measured at each patient's index visit. To account for the small sample size and non-normal distribution as confirmed by Shapiro–Wilk tests, non-parametric statistical testing was used. Demographic, clinical, and imaging data were compared between terciles using non-parametric Kruskal–Wallis tests. Variables identified as significant were subsequently evaluated with Dunn's pairwise multiple comparison tests to evaluate differences between cohorts. An alpha of 0.05 was selected as the significance threshold for all statistical tests. Data cleaning and statistical analyses were done using the R Statistical Software (R Version 2022.12.0 + 353, R Foundation for Statistical 136 Computing).

## Results

During our study period, 120 patients received index USE scans. The inclusion and exclusion criteria yielded 31 patients with index and follow-up USE scans. The median time between index and follow-up scans was 6.2 [6.0, 8.3] months. Most patients were of male sex and white race and had a pre-existing diagnosis of hypertension prior to the index scan. Table 1 summarizes key patient characteristics and comorbidities for the entire cohort.

**Table 1:** Baseline demographic characteristics of all patients included in the study at the time of the index scan.

Variable	Patient characteristics (*n* = 31)
	Median [IQR]/Frequency (%)
Age at baseline	72.0 [65.0, 77.5]
Age at follow-up	72.0 [65.0, 77.5]
Race, Caucasian	30 (96.8)
Sex, male	26 (83.9)
Hypertension	19 (61.3)
Active smoker	9 (29.0)
Type 2 diabetes mellitus	7 (22.6)
Atrial fibrillation	4 (12.9)
CKD	6 (19.4)
COPD	3 (9.7)
Neoplasm	7 (22.6)
Chronic anticoagulation	5 (16.1)
ACE-inhibitor	12 (38.7)
Statin	26 (83.9)
Beta-blocker	16 (51.6)
Tacrolimus	1 (3.2)
Cyclosporine	1 (3.2)

All continuous variables are described as median values with associated interquartile ranges (IQR).

### Evaluating changes in strain over time

**Figure 3:**
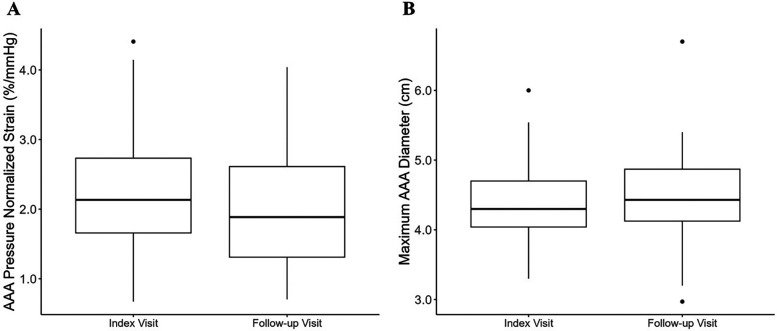
Box-and-whisker plot depicting the median $\bar{\varepsilon_{\rho +}}/\mathrm{PP}$ and maximum abdominal aortic aneurysm diameter across the entire study cohort at index and follow-up scan. The box plot depicts the median, first and third quartile values, and the whiskers depict the upper and lower extremes. Outliers are depicted as singular points on the graph. **(A)** Median AAA $\bar{\varepsilon_{\rho +}}/\mathrm{PP}$ at index and follow-up scan; **(B)** median maximum AAA diameter at index and follow-up scan.

Across the entire cohort, the maximum AAA diameter increased at a rate of 0.19 [−0.10, 0.47] cm/year, from a median of 4.3 [4.0, 4.7] cm at the index visit to 4.4 [4.1, 4.9] cm at follow-up (*p* = 0.04). The $\bar{\varepsilon_{\rho +}}/\mathrm{PP}$ decreased at a rate of −0.58 [−2.2, 0.27] %/mmHg/year, from 2.1 [1.7, 2.7] %/mmHg at index visit to 1.9 [1.3, 2.6] %/mmHg (*p* = 0.076). Figure 3 illustrates the changes in AAA diameter and $\bar{\varepsilon_{\rho +}}/\mathrm{PP}$ across the entire cohort.

An association between aneurysm growth rate and “strain tercile” has been described previously by our group, with aneurysms demonstrating “intermediate-strain” growing faster than “high” and “low-strain” aneurysms (27). This difference in growth rate was hypothesized to reflect the differing histopathologic processes occurring in each tercile. The cohort in the present study was divided into the terciles established in our prior work, using index $\bar{\varepsilon_{\rho +}}/\mathrm{PP}$ values of 0.025% and 0.038%/mmHg as cutoffs (27).

**Figure 4:**
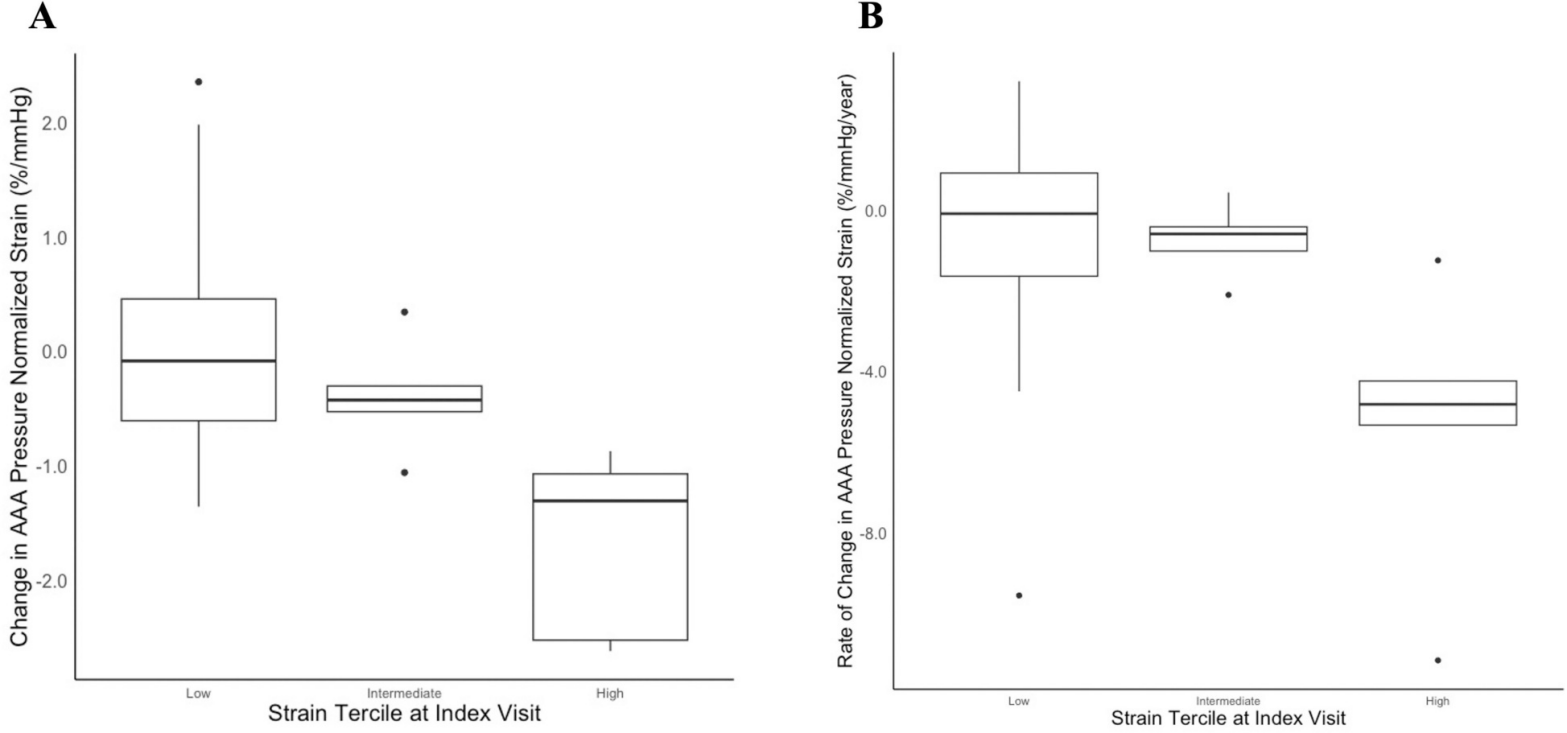
Box-and-whisker plot depicting change in AAA $\bar{\varepsilon_{\rho +}}/\mathrm{PP}\;\mathrm{from}$ index scan to follow-up scan stratified by patients’ strain tercile at the index visit. The box plot depicts the median, first and third quartile values, and the whiskers depict the upper and lower extremes. Outliers are depicted as singular points on the graph. **(A)** The net change in AAA $\bar{\varepsilon_{\rho +}}/\mathrm{PP}$ from index to follow-up scan; **(B)** the rate of change in AAA $\bar{\varepsilon_{\rho +}}/\mathrm{PP}$ per year as measured between index to follow-up scan.

Spearman's rank correlation test noted an association of −0.57 (*p* < 0.01) between the rate of $\bar{\varepsilon_{\rho +}}/\mathrm{PP}$ change and the index $\bar{\varepsilon_{\rho +}}/\mathrm{PP}$ measurement. The “high-strain” tercile was associated with a median $\bar{\varepsilon_{\rho +}}/\mathrm{PP}$ reduction of −1.3 [−2.5, −1.1] %/mmHg between index and follow-up imaging, as compared to the “low-strain” tercile (−0.082 [−0.61, 0.46] %/mmHg, *p* < 0.01) and “intermediate-strain” tercile (−0.42 [−0.53, −0.30] %/mmHg, *p* = 0.043) terciles (Figure 4A). The rate of $\bar{\varepsilon_{\rho +}}/\mathrm{PP}$ change in the high-strain tercile was −4.8 [−5.3, −4.2] %/mmHg/year vs. the low-strain tercile (−0.073 [−1.6, 0.93] %/mmHg/year, *p* = 0.004) and intermediate-strain tercile (−0.58 [−1.0, −0.40] %/mmHg/year, *p* = 0.047) (Figure 4B). There was no difference in patient demographics or comorbidities between terciles (Table 2).

**Table 2:** Baseline demographic characteristics of all patients included in the study at the time of the index scan, stratified by strain tercile at the index visit.

Variable	Patient characteristics			*p*-value
	Median [IQR]/Frequency (%)			
	Low-strain (*n* = 21)	Intermediate-strain (*n* = 5)	High-strain (*n* = 5)	
Age at baseline	74.00 [68.00, 78.00]	69.00 [63.00, 84.00]	65.00 [65.00, 68.00]	0.294
Age at follow-up	74.00 [68.00, 78.00]	70.00 [64.00, 84.00]	65.00 [65.00, 68.00]	0.247
Sex, male	18 (85.7)	4 (80.0)	4 (80.0)	0.921
Race, Caucasian	21 (100.0)	4 (80.0)	5 (100.0)	0.068
Hypertension	13 (61.9)	3 (60.0)	3 (60.0)	0.995
Active smoker	5 (23.8)	2 (40.0)	2 (40.0)	0.650
Type 2 diabetes mellitus	5 (23.8)	1 (20.0)	1 (20.0)	0.972
Atrial fibrillation	3 (14.3)	1 (20.0)	0 (0.0)	0.606
CKD	4 (19.0)	2 (40.0)	0 (0.0)	0.277
COPD	2 (9.5)	1 (20.0)	0 (0.0)	0.564
Neoplasm	5 (23.8)	0 (0.0)	2 (40.0)	0.310
Chronic anticoagulation	4 (19.0)	1 (20.0)	0 (0.0)	0.563
ACE-inhibitor	9 (42.9)	1 (20.0)	2 (40.0)	0.640
Statin	18 (85.7)	4 (80.0)	4 (80.0)	0.921
Beta-blocker	9 (42.9)	4 (80.0)	3 (60.0)	0.301
Tacrolimums	1 (4.8)	0 (0.0)	0 (0.0)	0.782
Cyclosporine	0 (0.0)	1 (20.0)	0 (0.0)	0.068

All continuous variables are described as median values with associated interquartile ranges (IQR).

### Proof of concept: correlating changes in strain over time with key clinical outcomes

Spearman's rank correlation tests showed no linear association between the rate of $\bar{\varepsilon_{\rho +}}/\mathrm{PP}$ change, AAA growth, rupture, intervention, or AAA-related mortality. These clinical outcomes were compared between patients who experienced an increase in $\bar{\varepsilon_{\rho +}}/\mathrm{PP}$ over time vs. a decrease in $\bar{\varepsilon_{\rho +}}/\mathrm{PP}$ (Table 3). The decreasing $\bar{\varepsilon_{\rho +}}/\mathrm{PP}$ cohort had an annual growth rate of 0.21 [−0.096, 0.79] cm/year vs. 0.17 [−0.12, 0.30] cm/year in the increasing $\bar{\varepsilon_{\rho +}}/\mathrm{PP}$ cohort though this was not significant (*p* = 0.42). One rupture and subsequent AAA-related mortality occurred in the decreasing $\bar{\varepsilon_{\rho +}}/\mathrm{PP}$ cohort, while none occurred in the increasing $\bar{\varepsilon_{\rho +}}/\mathrm{PP}$ cohort. Both groups had similar intervention rates at 1 year (29%/1 year in the decreasing strain cohort vs. 30%/1 year in the increasing strain cohort) and 5 years (62%/5 years vs. 60%/5 years).

**Table 3:** Key clinical outcomes in patients stratified by directionality of strain change from index to follow-up scan.

Variable	Strain change		*p*-value
	Median IQR/Frequency (%)		
	Decrease (*n* = 21)	Increase (*n* = 10)	
Growth (cm/year)	0.21 [−0.10, 0.79]	0.17 [−0.12, 0.30]	0.41
Rupture	1 (4.8)	0 (0.0)	1
1-Year intervention	6 (28.6)	3 (30.0)	1
5-Year intervention	13 (61.9)	6 (60.0)	1
1-Year AAA-related mortality	0 (0.0)	0 (0.0)	N.A.
5-Year AAA-related mortality	1 (4.8)	0 (0.0)	1

All continuous variables are described as median values with associated interquartile ranges (IQR).

## Discussion

The present study used USE to characterize changes in AAA $\bar{\varepsilon_{\rho +}}/\mathrm{PP}$ over time. The $\bar{\varepsilon_{\rho +}}/\mathrm{PP}$ decreased by 0.43% across the entire cohort (*p* = 0.076). This is consistent with multiple imaging-based *in-vivo* studies that have demonstrated that AAA walls are stiffer than normal aortic tissue (26, 29, 30). Furthermore, this finding aligns with the model of AAA degeneration proposed by Niestrawska et al., which predicts an increase in aneurysm wall stiffness and reduction in strain as collagen deposition occurs and a neo-adventitia forms in later-stage aneurysms. There was a concurrent increase in AAA diameter of 0.16 [−0.070, 0.23] cm (*p* = 0.042), but no linear association was observed between the changes in strain and changes in diameter.

The literature regarding the relationship between AAA diameter and the biomechanical properties of the aortic wall is conflicting. Studies published by Wilson et al. and van Disseldorp et al. report a positive correlation between AAA diameter and tissue stiffness (30, 31). However, studies by Long et al. and Dong et al. demonstrate no correlation between these two parameters (32, 33). With no consistent linear correlation reported between $\bar{\varepsilon_{\rho +}}/\mathrm{PP}$ and AAA diameter, it is likely that the relationship between AAA size and its biomechanical properties is multifactorial. When understood in the context of the histopathologic changes occurring in the aneurysm wall, the association between $\bar{\varepsilon_{\rho +}}/\mathrm{PP}$ and diameter changes is likely influenced by patient-specific remodeling patterns and the stage of degeneration at which these parameters are measured. This helps to explain the inconsistent reporting on how the biomechanical and morphometric properties of AAAs are correlated (27). For example, patients with similar AAA diameters may be at different stages in the inflammatory remodeling process, leading to discrepant elastin and collagen contents. The aortic walls of these patients would, therefore, have different biomechanical properties despite similar morphology. Without appropriately stratifying patients based on their stage of aneurysm progression, the utility of morphometric measures such as diameter and growth rate is limited as predictors of aneurysm rupture.

Dividing the cohort into terciles based on the index $\bar{\varepsilon_{\rho +}}/\mathrm{PP}$ allowed for separation and comparison of AAAs at different stages in the histopathologic remodeling process. The high-strain tercile demonstrated a statistically greater decrease in $\bar{\varepsilon_{\rho +}}/\mathrm{PP}$ as compared to the low and intermediate-strain terciles, implying a greater rate of stiffening of these AAAs over the follow-up period. The low-strain and intermediate-strain cohorts also decreased in strain but at significantly lower rates. These findings are consistent with the three-stage model for AAA degeneration effectively demonstrating the dynamic biological changes that occur in the aneurysm wall through biomechanical measurements captured via non-invasive imaging. The authors hypothesize that aneurysms that presented with high strain at the index visit had completed stage two of remodeling, characterized by inflammatory infiltration and increased wall compliance, and had transitioned to stage three by the follow-up visit with increased collagen deposition and formation of a neo-adventitia during the interval. The aneurysms that presented as low or intermediate strain terciles at the index visit were earlier in the remodeling process and experienced continued inflammatory cell-mediated ECM degradation during the follow-up period.

The differences in key clinical outcomes between strain terciles, including growth rate, surgical intervention, and time to repair, were evaluated in the study of the larger cohort by Zottola et al. (27). As the present study analyzed a smaller subset of the same study cohort, we did not repeat the comparison of clinical outcomes between strain terciles. Instead, we compared the clinical outcomes between AAAs that increased and decreased strain. We found no differences in growth, rupture, or intervention rates between these two groups. Two ruptures occurred in the group with decreasing strain, but this was not significant. Given the small sample size, a type 2 error may have occurred, and a relationship between strain changes and key clinical outcomes may be uncovered from a larger cohort study.

Aneurysmal degeneration, rupture, and the surgical decision to intervene are complex, multifactorial processes. This study aimed to characterize the natural history of AAA tissue mechanics *in vivo* using USE to understand how aneurysms degenerate over time. Our ultrasound imaging findings support the three-stage model for AAA degeneration proposed by prior histopathological studies, and demonstrate that non-invasive techniques such as ultrasound elastography can quantify changes in the AAA wall over time.

The development of novel tools and technologies to aid in the diagnosis and monitoring of AAAs is critical to improving the outcomes of this patient population. Furthermore, the technological landscape of medical imaging is constantly evolving. Novel displacement tracking algorithms based in regularized-optimization and deep learning-based methods may more reliably estimate vessel wall strain than non-rigid registration-based techniques such as ours (34–37). Although the feasibility of their implementation in routine clinical settings has yet to be determined, these techniques hold tremendous promise in advancing the role of aortic wall biomechanics in patient monitoring. The data derived from these technologies and techniques may 1 day inform physicians' clinical decision-making and allow them to tailor their treatment strategies to the unique disease process of each patient.

### Limitations

The small sample size and the short follow-up period may contribute to type 2 errors, considering the literature estimates the effect size of strain change in AAA to be small. The study's retrospective design and specific patient selection criteria, notably excluding patients who underwent endovascular aneurysm repair prior to follow-up, may introduce selection bias, as patients with clinically significant changes in their aneurysm morphology and biomechanics may have been excluded. The study population is also homogenous in that the participants were largely white males. These demographic characteristics limit the generalizability of our findings to patients not requiring intervention, and patients in other demographic sectors of the broader population. Furthermore, the findings of the present study were not cross-validated against other imaging modalities or evaluated for inter-operator reproducibility which limits their reliability. Future work should focus on expanding the cohort size and heterogeneity, extend follow-up length, and employ cross-validation techniques for more robust and generalizable data.

## Conclusion

The present study utilized a novel ultrasound elastography technique to characterize changes in AAA pressure-normalized wall strain over time, providing insight into the natural history of aneurysm wall tissue mechanics. The findings of this non-invasive technique is consistent with histopathologic models for aneurysm degeneration. This technique presents a promising avenue for improving the monitoring and management of AAAs, as evaluating biomechanical changes over time may help delineate the histologic progression of patients' disease independent of AAA diameter and growth rate.

## Data Availability

The datasets presented in this article are not made publicly available for the protection of patient privacy and HIPPA related information. Requests to access the datasets should be directed to Doran Mix, dmix@urmc.rochester.edu.
